# Genetic Variants in Potassium Channel Genes and Their Clinical Implications in Kazakhstani Patients with Cardiac Arrhythmias

**DOI:** 10.3390/jpm16020060

**Published:** 2026-01-26

**Authors:** Ayaulym Chamoieva, Saule Rakhimova, Zhannur Abilova, Ainur Akhmetova, Gulbanu Akilzhanova, Madina Zhalbinova, Asset Daniyarov, Kenes Akilzhanov, Askhat Molkenov, Ulykbek Kairov, Anargul Kuanysheva, Nurlan Shaimardanov, Ayan Abdrakhmanov, Makhabbat Bekbossynova, Ainur Akilzhanova

**Affiliations:** 1 Center for Life Sciences, National Laboratory Astana, Nazarbayev University, Astana 010000, Kazakhstan; ayaulym.chamoieva@nu.edu.kz (A.C.); zhannur.nurkina@nu.edu.kz (Z.A.); ainur.akhmetova2@nu.edu.kz (A.A.); madina.zhalbinova@nu.edu.kz (M.Z.); asset.daniyarov@nu.edu.kz (A.D.); askhat.molkenov@nu.edu.kz (A.M.); ulykbek.kairov@nu.edu.kz (U.K.); 2 Eurasian Society of Personalized Medicine, Astana 010000, Kazakhstan; 3 Department of Medicine, Semey Medical University, Pavlodar Branch, Pavlodar 140000, Kazakhstan; gulbanu.akilzhanova@smu.edu.kz (G.A.); kenes.akilzhanov@smu.edu.kz (K.A.); 4 Department of General Biology and Genomics, L. N. Gumilyov Eurasian National University, Astana 010000, Kazakhstan; 5 Medical Centre Hospital of the President’s Affairs Administration of the Republic of Kazakhstan, Astana 010000, Kazakhstan; a.g.kuanysheva@gmail.com; 6 Department of Medicine, Semey Medical University, Semey 071400, Kazakhstan; nurlan.shaimardanov@smu.edu.kz; 7 Heart Rhythm Institute, Medical University Astana, Astana 010000, Kazakhstan; abdrakhmanov.a@amu.kz; 8 Heart Center, Corporate Fund “University Medical Center”, Nazarbayev University, Astana 010000, Kazakhstan; mcardio_s@mail.ru; 9 The Academy of Public Administration Under the President of the Republic of Kazakhstan, Astana 010000, Kazakhstan

**Keywords:** cardiac arrhythmia, potassium channel genes, *KCNH2*, genetic testing, next-generation sequencing, sudden cardiac death, clinical genetics, Kazakhstan

## Abstract

**Background/Objectives**: Cardiac arrhythmias are among the leading causes of sudden cardiac death (SCD). Pathogenic variants in potassium channel genes play a key role in inherited arrhythmia syndromes, yet their contribution in Central Asian populations remains poorly characterized. **Methods**: We performed targeted next-generation sequencing (NGS) using a 96-gene custom Haloplex panel in 79 Kazakhstani patients with clinically diagnosed arrhythmias, including atrioventricular block, sick sinus syndrome, and atrial fibrillation. Detected variants in potassium channel genes were classified according to ACMG guidelines and correlated with clinical phenotypes. **Results**: A total of 52 variants were identified across 11 potassium channel genes. Two likely pathogenic variants (*KCNH2* p.Cys66Gly and p.Arg176Trp) and six variants of uncertain significance (VUS) in *KCNQ1*, *KCNE2*, *KCNE3*, and *KCNJ8* were detected. Two novel previously unreported variants were found in *KCNE5* and *KCND3.* Patients harboring pathogenic variants commonly presented with early-onset arrhythmias or a positive family history of cardiovascular disease. Carriers of *KCNH2* variants exhibited mild QT prolongation and recurrent syncope. **Conclusions**: This is the first genetic study of potassium channel gene mutations in Kazakhstani patients with cardiac arrhythmias. The detection of pathogenic and novel variants highlights the clinical utility of integrating genetic testing into diagnostic and management pathways for arrhythmia syndromes. Population-specific genomic data are essential for improving risk stratification, guiding medication safety, and enabling cascade family screening in Central Asia.

## 1. Introduction

Cardiovascular diseases (CVDs) remain the leading cause of mortality globally, accounting for approximately 18 million deaths in 2019 [1], with low- and middle-income countries such as Kazakhstan disproportionately affected. Cardiac arrhythmias are a major cause of morbidity and mortality worldwide, contributing significantly to sudden cardiac death (SCD) and heart failure. Despite advances in diagnostic and therapeutic strategies, a substantial proportion of arrhythmias remain idiopathic, with unclear underlying mechanisms [2,3]. Increasing evidence demonstrates that genetic variants, particularly in ion channel genes, are key determinants of both inherited and acquired arrhythmia susceptibility. Hereditary arrhythmia syndromes include primary electrical heart disorders, commonly referred to as cardiac channelopathies, caused by mutations in genes encoding cardiac ion channels [4]. Despite being asymptomatic, the channelopathies are associated with an increased risk of ventricular arrhythmias and SCD, highlighting the importance of early genetic diagnosis [5,6].

Among these, potassium channel genes play a pivotal role in regulating cardiac action potential repolarization. Notably, nearly 80 genes encode pore-forming subunits of potassium channels, representing the largest family of pore-forming channel proteins in the human genome. Pathogenic variants in *KCNQ1*, *KCNH2*, *KCNE1*, and related genes are established causes of long QT syndrome (LQTS), short QT syndrome (SQTS), Brugada syndrome (BrS), and atrial fibrillation (AF) [3,4,7,8]. Moreover, mutations in potassium channel genes can alter cardiac repolarization by disrupting normal ion flow across the cell membrane. Typically, loss-of-function mutations lead to delayed repolarization and are associated with LQTS, while gain-of-function mutations may shorten repolarization, contributing to AF and SQTS. Heterozygous missense variants are the most frequent and often disturb channel function through mechanisms such as protein misfolding, defective trafficking, or abnormal gating. Identification of the above-mentioned variants not only aids in confirming diagnosis but also informs risk stratification, pharmacogenomic guidance, and family screening [9,10,11].

To date, most genetic studies of arrhythmia syndromes have focused on European, East Asian, or North American populations. Data from Central Asia, including Kazakhstan, is scarce. Given the unique ethnic and genetic background of the Kazakh population, characterizing the local variant spectrum is critical for accurate interpretation and clinical translation of genomic findings [12,13]. The development of next-generation sequencing (NGS) has become a valuable tool for uncovering disease-causing variants, particularly when targeting specific genomic regions [14].

This study aimed to identify pathogenic and novel variants in potassium channel genes among Kazakhstani patients with clinically diagnosed cardiac arrhythmias using targeted NGS. We further sought to correlate genetic findings with clinical phenotypes and discuss implications for personalized arrhythmia management.

## 2. Materials and Methods

### 2.1. Study Population

The study was performed according to the principles of the Declaration of Helsinki. The research protocol was approved by the Nazarbayev University Institutional Research Ethics Committee—NU IREC (#866/18032024, protocol dated 19 April 2024). Written informed consent was obtained from all participants prior to inclusion in the study by the recruiting clinicians (co-authors), in accordance with the approved ethical protocol. To protect the rights of patients, their personal information was encoded, and all data were depersonalized during database collection. Patient recruitment was conducted between 2015 and 2016 during their treatment at the National Research Cardiac Surgery Center, NRCC (since 2023 Heart Center, Corporate Fund «University Medical Center», Nazarbayev University), Astana, Kazakhstan. DNA samples and sequencing data were generated and stored at NLA within the same ethical frame-work. The present manuscript represents a re-analysis of previously collected clinical and genomic data, and was performed within the scope of the approved ethics protocol. 

A total of 79 unrelated patients with arrhythmic disorders were included in our study group, including atrioventricular (AV) block, sick sinus syndrome (SSS), and atrial fibrillation (AF). Among AF cases, both idiopathic atrial fibrillation (iAF) and coronary artery disease-associated atrial fibrillation (CAD AF) were represented. iAF was defined as AF occurring in the absence of any structural heart disease, while CAD AF is AF developed as a result of coronary artery disease (angina pectoris (stenocardia), myocardial infarction, postinfarctional cardiosclerosis) confirmed by clinical records. The clinical diagnosis of arrhythmic disorders was based on the medical history of patients, clinical representation, and all instrumental findings and was made by clinicians. All study participants were recruited between 2015 and 2016 during their treatment at the National Research Cardiac Surgery Center, NRCC (since 2023 Heart Center, Corporate Fund «University Medical Center», Nazarbayev University), Astana, Kazakhstan. Demographics (age, gender, and ethnicity), family anamnesis and clinical features, results of the blood tests, electrocardiogram, 24 h Holter electrocardiogram monitoring, and echocardiogram were obtained from medical records (Supplementary Table S1). Moreover, 234 healthy Kazakh individuals from the population database of whole-genome sequencing were used as a control.

### 2.2. DNA Extraction

Peripheral blood samples were collected, and genomic DNA was extracted from whole blood using the Wizard^®^ Genomic DNA Purification kit (Promega, Madison, WI, USA) according to the manufacturer’s instructions. The quality (concentration and purity) of extracted DNA was measured using a NanoDrop™ spectrophotometer (Thermo Fisher Scientific, Waltham, MA, USA). The qualitative and quantitative analyses of samples were undertaken using the spectrophotometer Qubit 2.0 (Thermo Fisher Scientific, Waltham, MA, USA) and gel electrophoresis (2% agarose gel). The total volume of isolated DNA was 45 µL, and the concentration of DNA in 1 microliter was at least 5 ng/µL. Enrichment Control DNA was used as a control.

### 2.3. Targeted Sequencing

Targeted sequencing was performed using the Agilent SureSelect/Haloplex custom panel encompassing 96 genes associated with cardiac arrhythmias, including 11 potassium channel genes (Table 1).

DNA libraries for all individuals in our group were prepared using the HaloPlex Custom Panel Tier 1 kit (Agilent Technologies) according to the protocol “HaloPlex Target Enrichment System for Illumina Sequencing” (v.D5. May 2013). The protocol is available online (https://www.agilent.com). The Haloplex protocol is optimized for digestion of 225 ng of genomic DNA. Prepared DNA libraries were checked on the 2100 BioAnalyzer (Agilent Technologies, Santa Clara, CA, USA) for quality using the High Sensitivity DNA Assay kit (Thermo Fisher Scientific). Then, pooled samples were loaded onto the Illumina HiSeq 2000 platform (San Diego, CA, USA) for sequencing. The design was adapted to the paired-end 150 bp sequencing technology. The size of target region made up 463.767 kbp. Concerning the realization of the NGS technology and the accuracy of sequences, the mean target coverage was 99.46%. We considered that it was correctly covered and then suitable for further data analysis.

### 2.4. Bioinformatics and Variant Annotation

Raw data were processed using standard bioinformatics pipelines (BWA (v0.7.18) for alignment, GATK (v4.3.0.0) for variant calling). Genetic variants were analyzed using the online software SureCall Design (Agilent Technologies, Santa Clara, CA, USA). The obtained variants corresponded to the Human Gene Mutation Database (HGMD) and were annotated with ANNOVAR (release 24 October 2019). The clinical significance of the variants was interpreted in accordance with the guidelines developed by the American College of Medical Genetics and Genomics (ACMG) and the Association of Molecular Pathology (AMP) in 2015 and classified as pathogenic (P), likely pathogenic (LP), a variant of uncertain significance (VUS), likely benign (LB), or benign (B) [15]. Additionally, the obtained variants were checked in online databases of Clinvar (http://www.ncbi.nlm.nih.gov/clinvar, accessed on 22 September 2025) and Franklin Genoox (https://franklin.genoox.com/). Population frequencies were verified against international research databases, the 1000 Genomes (1000G), the Genome Aggregation Database (gnomAD), Exome Aggregation Consortium (ExAC), Online Mendelian Inheritance in Man (OMIM), Mutation Taster, NCBI, etc. Additionally, allele frequencies of variants were compared with a cohort of 234 healthy Kazakh individuals.

The control whole-genome sequencing dataset was generated using the DNBSEQ-G400 platform (BGI) with a mean genome-wide coverage of approximately 34X.

Variant pathogenicity was assessed using SIFT (version 1.3.0) and PolyPhen-2 (12 January 2024) and CADD phred (1.1.0).

All sequence data presented in this study are deposited in the NCBI Sequence Read Archive (SRA) repository and are publicly available under accession BioProject number PRJNA908657 (https://www.ncbi.nlm.nih.gov/bioproject/?term=PRJNA908657, accessed on 10 October 2025).

### 2.5. Statistical Analysis

Statistical analysis was performed using the IBM SPSS Statistics 23.0 program (SPSS, Chicago, IL, USA). Continuous variables were presented as mean ± standard deviation, and categorical variables as numbers (n) or percentages (%).

## 3. Results

### 3.1. Clinical Characteristics

The cohort of 79 patients included the following most common diagnoses: atrial fibrillation (59.5%), AV block (20%), and sick sinus syndrome (20%). The clinical characteristics of the patients are provided in Table 2.

The mean age at onset in the study group was 47.5 ± 17.5 years; five patients (6%) were under 18 years of age. The cohort included 52 (65.8%) male patients. The research group consisted of Kazakhs (64.2%), Russians (17.3%), and other nationalities (18.5%). Additionally, 14 (18%) of the patients had a prolonged QT interval (QT ≥ 440 ms), and 12 (15%) reported experiencing syncope in their medical history. Of 79 patients with arrhythmias, 23 individuals (29%) had a predisposition to cardiovascular diseases in their family history. Due to serious cardiac risks accompanying life-threatening arrhythmias, 31 (39%) patients underwent pacemaker implantation, namely almost all of the AV block and SSS patients. The average body mass index (BMI) of the patients in the cohort was 26.8 ± 5.4 kg/m^2^, and 11 (14%) patients had type 2 diabetes. In addition, nine patients with CAD AF had suffered a myocardial infarction. None of the patients had a clinical death (cardiac arrest).

### 3.2. Genetic Findings

A total of 52 variants were identified across 11 potassium channel genes; full details are provided in Supplementary Table S2. According to the ACMG classification [15], these included two likely pathogenic variants in *KCNH2* (p.Cys66Gly, p.Arg176Trp) and six VUS in *KCNQ1*, *KCNE2*, *KCNE3*, and *KCNJ8* (Table 3).

The variants can be considered very rare with a minor allele frequency (MAF) threshold of ≤0.02% in all used population databases (gnomeAD, ExAC, dbSNP).

Categorization of all potassium channel genes according to ACMG classification indicated that the number of mutations in the iAF study group is higher than in other groups, as well as the number of patients. AV block, SSS, and CAD AF patients share almost the same number of genetic variants in potassium channel genes.

Notably, a prolonged QT interval (>440 ms) was not observed in any patient carrying disease-causing variants.

Overall, 17 variants in potassium channel genes were presented in the HGMD database (accessed August 2025, Table 4).

Among them, seven variants had previously been reported as disease-causing (DM) in the HGMD, and two additional variants (*KCNE2* p.Thr10Met and *KCNE5* p.Tyr81His) were labeled as probable disease mutations (DM?), indicating uncertain but possible pathogenicity. Two variants (*KCNA5* p.Pro307Ser and *KCNH2* p.Arg1047Leu) were categorized as disease-causing with functional proof (DFP). Furthermore, five variants were listed as functionally relevant polymorphisms (DF/FP), yet were not considered clinically pathogenic, including *KCNH2* p.Lys897Thr and *KCNE1* p.Ser38Gly, which showed relatively high allele frequencies (>0.01) in gnomAD and were marked as benign in ClinVar.

In this study, we identified two variants in *KCND3*, *KCNE5* that were not present in the dbSNP, gnomAD, ClinVar, and HGMD databases, suggesting they may represent novel or extremely rare variants (Table 5).

The frequency filtering was performed by comparing two unreported variants against a control cohort of 234 healthy Kazakh individuals to assess population-specific novelty. As a result, these variants were not observed in the control group. Also, the variants were evaluated using in silico prediction tools. *KCND3* p.Pro643His showed a high CADD phred score (≥20), consistent with potential deleteriousness. *KCNE5* p.Gln126His yielded mixed or tolerated predictions across SIFT PolyPhen-2 and CADD phred.

### 3.3. Genotype–Phenotype Correlations

Possibly disease-causing variant carriers (*n* = 11, Table 3) and non-carriers (*n* = 68) were assessed statistically on the QT interval parameter (Supplementary Table S3). QT intervals were numerically shorter in variant carriers compared with non-carriers (375.7 ± 25.7 ms vs. 389.8 ± 66.2 ms); however, this difference did not reach statistical significance (ANOVA, F = 0.48, *p* = 0.49). Levene’s test indicated heterogeneity of variances (*p* = 0.018).

Statistically significant clinical parameters (*p* < 0.05) of patients were analyzed by the Kruskal–Wallis test in Table 6.

Using the Kruskal–Wallis test, no statistically significant differences were observed in the QT interval between the CAD AF and iAF groups (*p* = 0.946). In contrast, other clinical parameters, including LVEF, LV EDD, LV ESD, and LA, showed statistically significant differences in pairwise comparisons. Furthermore, significant differences across all assessed parameters were observed between the AV block–CAD AF and SSS–CAD AF groups.

## 4. Discussion

We evaluated the contribution of genetic variants in potassium channel genes in a cohort of 79 patients with cardiac arrhythmias. Particularly, we used a targeted NGS panel consisting of 96 genes relevant to arrhythmias and cardiomyopathies to overcome the limitations of unselective sequencing. Despite the broader scope of whole-genome/whole-exome sequencing, a targeted gene enrichment approach provides full coverage, high accuracy, and high sensitivity for specific genes [16].

In our study, the most commonly noted LQTS-causing variants were found in the *KCNH2* and *KCNQ1* genes, which encode the α subunits of potassium channels Kv11.1/hERG and Kv7.1/KvLQT1, respectively. Nearly 75% of LQTS disorder is caused by the main LQTS genes—*KCNQ1*, *KCNH2*, and *SCN5A* [17,18]. Around 30–35% patients with LQTS have mutations in *KCNQ1*, while 25–40% have mutations in *KCNH2*, highlighting the clinical significance of potassium channels and their inclusion in diagnostic panels [19]. Namely, we observed 13 mutated alleles of the *KCNH2* gene and 8 variants representing the *KCNQ1* gene. These genes are responsible for LQTS type 1 and LQTS type 2, respectively.

Our study demonstrates that genetic variants in potassium channel genes are present in a significant proportion of Kazakhstani patients with arrhythmias, even in the absence of overt QT prolongation or structural heart disease. This underscores the value of genetic testing as an adjunct to clinical evaluation, particularly in idiopathic arrhythmias or early-onset AF. Approximately 18% of the patients in our cohort exhibited prolonged QT, and 15% reported syncope—both key risk markers for SCD. Normal QT is considered ≤440 ms for men and ≤460 ms for women. QT > 450/470 ms indicates prolongation; ≥480 ms is used diagnostically in symptomatic individuals, and ≥500 ms confers high risk [10,18,20]. However, none of the patients carrying disease-causing variants showed QT interval prolongation in our cohort. Recent studies show that a significant number of carriers of pathogenic LQTS gene variants have a baseline QT within the normal range. In genotyped LQTS patients, approximately half of them have no lifetime symptoms, and from 10 to 50% observe no visible QT prolongation in an electrocardiographic pattern [18,21,22]. Although possibly disease-causing variant carriers tended to have shorter QT intervals in our study, the difference was not statistically significant, likely due to the small number of carriers and high variability in QT duration. Among study participants, a patient (#89) diagnosed with AV block (III stage) has LQTS (QT = 506 ms), including symptoms of syncope. Also, the patient’s diagnosis is mediated by ventricular arrhythmia. However, disease-causing variants in potassium channel genes were not observed.

Glazer et al. reported strong associations between pathogenic/likely pathogenic variants in *KCNQ1*, *KCNH2*, and *KCNE1* and arrhythmias (adjusted ORs 20–25) in an unselected population cohort. Importantly, Glazer and colleagues used a PM2 threshold of MAF < 0.00005 (0.005%) in gnomAD when assessing rarity for arrhythmia syndromes, including LQTS [23]. Notably, among the rare variants identified in our cohort, the VUS *KCNQ1* c.1128+4C>T (gnomAD MAF 0.00001) met the PM2 rarity cutoff (PM2 = ACMG/AMP criterion for absence or extreme rarity in population databases [15]).

In our study, among 52 variants in 11 potassium channel genes, particularly, variants with pathogenicity status were found in *KCNH2* (p.Cys66Gly, p.Arg176Trp), *KCNQ1* p.Tyr662Ter, and *KCNE3* p.Thr4Ala. According to ACMG classification, patients #516, #334, and #377 carry a rare mutation in a likely pathogenic variant *KCNH2* p.Cys66Gly, NM_000238.4):c.196T>G. The variant is located within the PAS domain, a critical regulatory segment of the HERG (KCNH2) channel that governs inactivation of Ikr current. As described by Gustina et al. (2012), mutations in this domain, particularly within residues 26–135, impair Ikr inactivation and promote delayed repolarization [24]. Patient #516 with CAD AF had suffered a myocardial infarction, reduced LVEF (44%), and a familial predisposition to CVD, which suggests a potential modifier effect, potentially associated with arrhythmogenic risk despite the absence of evident LQTS. A recent population-based cohort study confirmed a strong association between family history and increased occurrence of AF [25]. The presence of AF in relatives approximately doubles the risk of AF and is associated with an earlier disease onset [26]. Consistent with this, the number of disease-associated variants is highest among individuals with AF onset before age 30 [27]; accordingly, our patients (#377, #282, #334) with AF before age 30 fit this pattern of hereditary early-onset AF. Our findings demonstrate that clinically relevant variants in potassium channel genes are detectable in a considerable proportion of Kazakhstani patients with arrhythmias, supporting the growing recognition that ion channel dysfunction contributes not only to classical LQTS or BrS but also to more common arrhythmia phenotypes such as AF and conduction disorders. Patient # 334 with iAF has a manifesting paroxysmal orthodromic AV-reentry tachycardia and Wolff–Parkinson–White (WPW) syndrome. The latter is associated with increased risk of supraventricular tachyarrhythmia and SCD [28]. According to ACMG classification, both patients (#202 and #573) carry a likely pathogenic variant *KCNH2* p.Arg176Trp (rs36210422), class 4 (LP). Notably, both individuals presented with paroxysmal forms of AF, had no documented family history, and demonstrated normal LVEF (64.7%, 52%). Nevertheless, the allele frequency in gnomAD is quite low, 0.000618 (≈0.062%), which indicates that the variant is extremely rare in the general population and may be consistent with a potential disease association. Rare variants in ion channel genes, including potassium channels, have been linked to monogenic forms of AF through mechanisms that alter atrial refractoriness, action potential duration, or intercellular conduction [29]. Such electrophysiological disturbances may explain the occurrence of AF itself. Despite the absence of structural heart disease or QT prolongation, the presence of this variant in two unrelated cases supports its potential pathogenic role in arrhythmogenesis. A recent large-scale cohort study confirmed that *KCNH2* p.Arg176Trp was significantly enriched among individuals referred for LQTS genetic testing, particularly those with no identified genetic cause, suggesting that this variant may act as a risk-modifying allele under specific triggers such as medication or stress [30].

Based on the HGMD database, several rare variants were found in key potassium channel genes (such as *KCNQ1* Tyr662Ter and *KCNH2* p.Tyr652Ter, p.Cys66Gly, p.Arg176Trp), which have been previously reported in association with arrhythmia phenotypes. (Table 4). Although ClinVar shows mixed interpretations, their classification as disease-causing in HGMD and their very low frequency in the population (gnomAD < 0.001) suggest that these variants may be potentially associated with disease. Two additional variants (Pro307Ser in *KCNA5* and Arg1047Leu in *KCNH2*) have been labeled as functionally abnormal, DFP, strengthening their clinical importance. Other variants (Lys897Thr and Ser38Gly) with DF/FP status, are likely benign but could still influence risk depending on the patient’s genetic background. Interestingly, several of the identified variants in our cohort, including *KCNQ1* p.Tyr662Ter, *KCNH2* p.Tyr652Ter, and *KCNE3* p.Thr4Ala, have been reported in previous studies as disease-causing mutations primarily in European or East Asian populations [31,32]. However, their detection in Central Asian cohort highlights the potential for both shared and population-specific genetic architectures of arrhythmic disorders. This underscores the importance of expanding genetic studies to include underrepresented populations, as founder effects and local allele frequencies may influence variant interpretation and clinical management. Two novel variants detected in *KCNE5* and *KCND3* may represent population-specific polymorphisms or low-frequency pathogenic alleles. Establishing regional genomic reference data is essential for accurate variant interpretation under ACMG criteria and to prevent misclassification of benign ethnic-specific variants.

Additionally, VUS was detected in *KCNQ1*, *KCNE2*, *KCNE3*, *KCNJ8*. Although the pathogenicity of these variants requires further functional validation, prior studies indicate that up to 40–45% of VUS in ion channel genes may exhibit functional defects [33]. Notably, four out six patients carrying VUS have a family history predisposition to CVD. VUS and DFP variants may be relevant depending on the clinical context and additional functional analysis. Thus, in symptomatic or familial cases, VUS should not be disregarded but rather integrated into risk assessment models and periodically re-evaluated as population data evolve.

Both novel variants affect residues located in conserved regions across vertebrate species, suggesting potential functional relevance. The KCND3 p.Pro643His variant resides in the C-terminal region of the Kv4.3 channel, which has been implicated in channel regulation and trafficking. Substitution of a structurally rigid proline with a histidine may alter local conformational stability or regulatory interactions. The KCNE5 p.Gln126His variant is positioned near the transmembrane region of the OI subunit, which modulates potassium channel gating. Replacement of glutamine with histidine may influence subunit interaction or channel kinetics, potentially affecting electrophysiological properties.

It is important to mention that as multigene panels become more widely used, rare variants are increasingly found in individuals without clear disease. Mutation databases are full of benign/likely benign variants that were previously known to be disease-causing; consequently, interpretation of genetic variants is quite complicated. Therefore, in our study, we systematically reannotated identified variants and updated their classifications by cross-checking population databases.

As outlined in the 2022 European Society of Cardiology (ESC) guidelines and the Heart Rhythm Society (HRS) consensus statement, genetic testing is recommended for patients with unexplained syncope, idiopathic ventricular tachycardia, or family history of SCD [16]. Integrating genetic results into cardiology practice offers several benefits. Genetic variants in cardiac ion channel genes, particularly *KCNH2* and *KCNQ1* associated with LQTS, significantly increase susceptibility to drug-induced QT prolongation. In such individuals, exposure to QT-prolonging medications, including class I/III antiarrhythmics, macrolide and fluoroquinolone antibiotics, antipsychotics, and antidepressants, may precipitate malignant ventricular arrhythmias, including torsades de pointes. Reduced repolarization reserve caused by pathogenic variants can render even modest pharmacological effects clinically significant. In addition, polymorphisms in drug-metabolizing enzymes (e.g., *CYP2D6*, *CYP3A4*) may elevate drug plasma levels, further amplifying proarrhythmic risk [34]. These observations highlight the importance of integrating pharmacogenomic data into clinical decision making to guide drug selection, dosing, and monitoring, thereby advancing the implementation of personalized cardiovascular medicine [10,35,36]. Importantly, at-risk relatives of affected patients are advised to undergo family cascade testing for early detection of carrier variants. Our findings provide empirical support for extending these recommendations to the Central Asian population.

Several limitations of this study should be acknowledged. First, the relatively small cohort size (79 patients) limits the generalizability and statistical power to detect genotype–phenotype correlations or estimate variant frequencies with precision. Moreover, stratified analysis by ethnicity was not performed because the small number of cases in each clinical subgroup of patients limited the ability to assess genetic heterogeneity. Second, although we used a comprehensive 96-gene targeted NGS panel providing high coverage and sensitivity, this approach does not detect structural variants, deep intronic changes, large copy number alterations, or variants in genes outside the panel that may also contribute to arrhythmogenic phenotypes. Third, segregation analysis was not systematically performed in affected families, which restricts the ability to confirm variant pathogenicity through inheritance patterns. Fourth, functional validation of the identified variants, particularly those of uncertain significance (VUS) and novel findings, was not conducted; therefore, their predicted effects remain speculative. Fifth, QT interval measurements and clinical data were collected at a single time point, and longitudinal follow-up could provide additional insight into variable expressivity or age-related penetrance. Finally, population-specific allele frequencies in Central Asian groups remain poorly characterized, which complicates the interpretation of variant rarity and potential founder effects. We are performing a large-scale population study in Kazakhstan to establish the Kazakhstani Reference Database of genetic variants. Future studies with larger multiethnic cohorts, extended family analyses, and electrophysiological characterization of novel variants are warranted to validate and expand upon these findings.

## 5. Conclusions

This study provides the first comprehensive assessment of potassium channel gene variants in Kazakhstani patients with cardiac arrhythmias. Identification of pathogenic and novel variants demonstrates the genetic contribution to arrhythmia pathogenesis and underscores the clinical utility of NGS-based testing. Integration of genetic diagnostics into cardiology practice can improve patient stratification, optimize therapy, and enable family-based prevention strategies in Central Asia.

We identified several clinically significant variants and two previously unreported potassium channel variants in a cohort of 79 patients with cardiac arrhythmias using a 96-gene targeted NGS panel (Haloplex). None of these novel variants were detected in 234 healthy Kazakh controls. Variant classification followed ACMG/AMP criteria, and unreported variants were further assessed using multiple in silico prediction tools; however, only a subset met rarity thresholds in population databases. These results highlight the utility of high-coverage targeted gene panels for detecting rare, potentially population-specific ion channel variants. To establish their pathogenicity and clinical relevance, segregation analyses and in vitro functional validation using patient-derived iPSC cardiomyocyte models are warranted. Functional validation of novel variants and family-based segregation analysis will be crucial next steps toward implementing precision cardiology in Central Asia.

## 6. Patents

Patent of the Republic of Kazakhstan for Utility Model No. 4574. Method for detecting genetic predisposition to cardiac arrhythmias based on a developed cardiogenetic panel of 96 candidate genes/Akilzhanova A.R., Abilova Zh.M., Akhmetova A.Zh., Kozhamkulov U.A., Rakhimova S.E., Kairov U.E. Published on 26 December 2019, application No. 2019/0880.2 dated 11 October 2017.

## Figures and Tables

**Table 1:** Selected potassium channel genes associated with arrhythmia.

Gene	Protein	Chromosome	Transcript	OMIM	Phenotype
*KCNA5*	Kv1.5	12p13.32	NM_002234	176267	AF
*KCND3*	Kv4.3	1p13.2	NM_172198	605411	BrS
*KCNE1*	Kv7.1	21q22.12	NM_000219	176261	LQTS, Jervell and Lange Nielsen syndrome
*KCNE2*	Kv7.2	21q22.11	NM_172201	603796	LQTS, AF
*KCNE3*	Kv7.3	11q13.4	NM_005472	604433	BrS
*KCNE5*	Kv7.5	Xq23	NM_012282	600681	BrS
*KCNH2*	Kv11.1/hERG	7q36.1	NM_172057/NM_000238	152427	LQTS, SQTS
*KCNJ2*	Kir2.1	17q24.3	NM_000891	600681	Andersen–Tawil syndrome, SQTS, AF
*KCNJ5*	Kir 3.4	11q24.3	NM_000890	600734	LQTS
*KCNJ8*	Kir 6.1	12p12.1	NM_00498	600935	BrS, SQTS, IVF associated, ERS
*KCNQ1*	Kv7.1	11p15.5-p15.4	NM_181798	607542	LQTS, SQTS, AF, Jervell and Lange Nielsen syndrome

AF, atrial fibrillation; BrS, Brugada syndrome; LQTS, long QT syndrome; SQTS, short QT syndrome; IVF, idiopathic ventricular fibrillation; ERS, early repolarization syndrome.

**Table 2:** Patient characteristics.

Characteristics	Total, *n* = 79	AV Block, *n* = 16	SSS, *n* = 16	iAF, *n* = 31	CAD AF, *n* = 16
Age, years	47.5 ± 17.5	45.6 ± 23.8	47.7 ± 17.6	42.7 ± 13.9	58.4 ± 11.9
Gender, M/F	52/27	6/10	9/7	22/9	15/1
BMI, kg/m^2^	26.8 ± 5.4	26.1 ± 6	25.5 ± 5.4	27.3 ± 5.3	28.2 ± 5.4
Family history CVD, N	23 (29%)	3	4	8	8
Diabetics, N	11 (14%)	2	1	5	3
Syncope, N	12 (15%)	7	4	0	1
Pacemaker implantation, N	31 (39%)	14	16	0	1
Prolonged QT, N	14 (18%)	5	6	0	3

**Table 3:** List of patients with rhythm disorders carrying rare possibly disease-causing variants.

Case ID	Sex	Age	Group	Clinical Phenotype	LVEF (%)	QT Interval, ms	Family History	Gene	Nucleotide	AA Change	Genotype	gnomeAD	Exonic Effect	HGMD	ACMG Score
#516	M	55	CAD AF	Myocardial infarction	44	378	yes	*KCNH2*	NM_000238.3:c.196T>A	p.Cys66Gly	T/T hom	-	missense	DM	4
#334	F	20	iAF	WPW syndrome, Paroxysmal orthodromic AV-reentry tachycardia	61	402	no	*KCNH2*	NM_000238.3:c.196T>A	p.Cys66Gly	T/T hom	-	missense	DM	4
#377	M	24	iAF	A blood thrombus in the auricle of the left atrium	65	320	no	*KCNH2*	NM_000238.3:c.196T>A	p.Cys66Gly	T/T hom	-	missense	DM	4
#202	M	76	iAF	CHF I, Paroxysmal AF	65	340	no	*KCNH2*	NM_000238.3:c.526C>T	p.Arg176Trp	G/A het	0.000618	missense	DM	4
#573	M	41	iAF	EHRA II. CHF, NYHA I, Paroxysmal AF	52	392	no	*KCNH2*		p.Arg176Trp	G/A het	0.000618	missense	DM	4
#464	F	30	iAF	Paroxysmal AF, EHRA I. Left atrial flutter, Atrial extrasystole	58	380	no	*KCNE2*	NM_172201.1:c.29C>A	p.Thr10Lys	C/A het	0.000968	missense	DM?	3
#80	M	57	SSS	Arterial hypertension	60	−390	yes	*KCNE3*		p.Thr4Ala	T/C het	0.000646	missense	DM	3
#282	M	22	iAF	Paroxysmal AF	-	400	no	*KCNQ1*	NM_000218.2:c.1128+4C>T	-	C/T het	0.00001	intron	N/A	3
#93	F	47	AV block	Arterial hypertension	66	−385	yes	*KCNQ1*	NM_000218.2:c.1033-4C>T	-	C/T het	-	intron	N/A	3
#333	M	30	iAF	CHF NYHA II, persistent AF	46	360	yes	*KCNJ8*	NM_004982.3:c.263C>G	p.Ala88Gly	G/C het	-	missense	N/A	3
#579	M	64	CAD AF	CHF NYHA II, EHRA I. Persistent AF	36	386	yes	*KCNJ8*	NM_004982.3:c.1145A>G	p.Lys382Arg	T/C het	-	missense	N/A	3

LVEF, left ventricular ejection fraction; AA, amino acid; CHF, congestive heart failure; DM, disease-causing mutation.

**Table 4:** Potassium channel gene variants in HGMD.

Gene	HGMD Mutation	Variant Class	dbSNP Identifier	gnomeAD	ClinVar
*KCNH2*	Tyr652Ter	DM	rs1137617	-	Not provided
*KCNQ1*	Tyr662Ter	DM	rs1161907	0.000649	Conflicting interpretations of pathogenicity
*KCNH2*	Cys66Gly	DM	rs199473416	-	Not provided
*KCNE3*	Thr4Ala	DM	rs200856070	0.000646	Conflicting interpretations of pathogenicity
*KCNQ1*	Lys393Asn	DM	rs12720457	0.000387	Conflicting interpretations of pathogenicity
*KCNH2*	Arg176Trp	DM	Rs36210422	0.000618	Conflicting interpretations of pathogenicity
*KCNE2*	Thr10Met	DM?	rs199473648	0.000968	Conflicting interpretations of pathogenicity
*KCNE5*	Tyr81His	DM?	Rs199924386	0.000324	Benign
*KCNQ1*	Pro448Arg	DM?	Rs12720449	0.005302	Benign/Likely benign
*KCNA5*	Pro307Ser	DFP	Rs17215409	0.002235	Conflicting interpretations of pathogenicity
*KCNH2*	Arg1047Leu	DFP	Rs36210421	0.026377	Conflicting interpretations of pathogenicity
*KCNH2*	Lys897Thr	DFP	Rs1805123	0.185003	Benign
*KCNE1*	Ser38Gly	DFP	rs1805127	0.659157	Benign
*KCNJ2*	Leu382Leu	DF	Rs173135	0.118301	Benign
*KCNE3*	Phe66Phe	DF	rs2270676	0.138846	Benign
*KCNJ5*	Glu282Gln	FP	rs7102584	0.015136	Benign

DM, disease-causing mutation; DFP, disease-causing mutation with functional proof; DF, disease-associated polymorphism with functional evidence; FP, in vitro/laboratory or in vivo functional polymorphism.

**Table 5:** Novel (previously unreported) variants in potassium channel genes.

Case ID	Gene	Transcript	Nucleotide	Protein Change	Geno Type	Chr	Exonic Effect	SIFT Score	Polyphen-2 Score	CADD Phred
472	*KCND3*	NM_172198	c.1928C>A	p.Pro643His	0/1	1	missense	0.8741, tolerated	0.961, probably damaging	22.5, deleterious
513	*KCNE5*	NM_012282	c.378G>T	p.Gln126His	0/1	X	missense	0.7802, tolerated	0.138, possibly damaging	8.025, tolerated

SIFT, Sorting Intolerant From Tolerant; CADD, Combined Annotation Dependent Depletion.

**Table 6:** Statistical testing of clinical parameters (Kruskal–Wallis test).

	Study Group	Median	25%	75%	*p* Value	Pairwise Comparison	*p* Value
QT interval, ms	AV block SSS CAD AF iAF	440 460 362 360	400 429 330 334	490 495 388 392	0.001	AV block–SSS AV block–CAD AF AV block–iAF SSS–CAD AF SSS–iAF CAD AF–iAF	0.946 0.002 * 0.000 * 0.008 * 0.001 * 0.957
LVEF, %	AV block SSS CAD AF iAF	66.5 61.5 38.2 58	59 60 28 55	67 69 50 64.7	0.000	AV block–SSS AV block–CAD AF AV block–iAF SSS–CAD AF SSS–iAF CAD AF–iAF	0.781 0.000 * 0.116 * 0.000 * 0.632 0.000 *
LV EDD	AV block SSS CAD AF iAF	47.5 49 59 48	43 44 52 42	52 53 63 52	0.004	AV block–SSS AV block–CAD AF AV block–iAF SSS–CAD AF SSS–iAF CAD AF–iAF	0.915 0.000 * 0.563 0.000 * 0.941 0.000 *
LV ESD	AV block SSS CAD AF iAF	31 32 46 33	26 28 40 31	36 37 53 39	0.000	AV block–SSS AV block–CAD AF AV block–iAF SSS–CAD AF SSS–iAF CAD AF–iAF	0.655 0.000 * 0.111 0.000 * 0.766 0.000 *
LA	AV block SSS CAD AF iAF	34.5 31 45 35	29 29 43 32	37 38 50 39	0.025	AV block–SSS AV block–CAD AF AV block–iAF SSS–CAD AF SSS–iAF CAD AF–iAF	0.945 0.000 * 0.462 0.000 * 0.165 0.000 *

LVEF—left ventricle ejection fraction, LA—left atrial dimension, LV EDD—left ventricular end-diastolic dimension, LV ESD—left ventricular end-systolic dimension. *—statistically significant.

## Data Availability

All sequence data presented in this study are deposited in the NCBI Sequence Read Archive (SRA) repository and are publicly available under accession BioProject number PRJNA908657 (https://www.ncbi.nlm.nih.gov/bioproject/?term=PRJNA908657, accessed on 10 October 2025). The data presented in this study are available on request from the corresponding author. The data are not publicly available due to ethical and privacy restrictions indicated in informed consent.

## Supplementary Materials

The following supporting information can be downloaded at: https://www.mdpi.com/article/10.3390/jpm16020060/s1, Table S1: Clinical characteristics of patients; Table S2: List of genetic variants in potassium channel genes; Table S3: The comparison of QT intervals between carriers and non-carriers.

## Abbreviations

The following abbreviations are used in this manuscript:

CVD	Cardiovascular diseases
NGS	Next-generation sequencing
SCD	Sudden cardiac death
LQTS	Long QT syndrome
SQTS	Short QT syndrome
BrS	Brugada syndrome
AF	Atrial fibrillation
CAD	Coronary artery disease
HGMD	Human Gene Mutation Database
ACMG	American College of Medical Genetics and Genomics
